# A hybrid technique to enhance the rainfall-runoff prediction of physical and data-driven model: a case study of Upper Narmada River Sub-basin, India

**DOI:** 10.1038/s41598-024-77655-5

**Published:** 2024-11-01

**Authors:** Sachin Kumar, Mahendra Kumar Choudhary, T. Thomas

**Affiliations:** 1 Department of Civil Engineering, Maulana Azad National Institute of Technology, Bhopal, 462003 India; 2 National Institute of Hydrology, Bhopal, 462003 India

**Keywords:** Rainfall-runoff, Physical models – WEAP, Data-driven models – ANN, Sensitivity analysis, Novel aproach - ANN_Hybrid_, Environmental sciences, Hydrology, Engineering

## Abstract

Accurate streamflow prediction is crucial for effective water resource management and planning. This study aims to enhance streamflow simulation accuracy in the data-scarce Upper Narmada River Basin (UNB) by proposing a novel hybrid approach, ANN_Hybrid_, which combines a physically-based model (WEAP) with a data-driven model (ANN). The WEAP model was calibrated and validated using observed streamflow data, while the ANN model was trained and tested using meteorological variables and simulated streamflow. The ANN_Hybrid_ model integrates simulated flow from both WEAP and ANN to improve prediction accuracy. The results demonstrate that the ANN_Hybrid_ model outperforms the standalone WEAP and ANN models, with higher NSE values of 95.5% and 92.3% during training and testing periods, respectively, along with an impressive R^2^ value of 0.96. The improved streamflow predictions can support better decision-making related to water allocation, reservoir operations, and flood and drought risk assessment. The novelty of this research lies in the development of the ANN_Hybrid_ model, which leverages the strengths of both physically-based and data-driven approaches to enhance streamflow simulation accuracy in data-limited regions. The proposed methodology offers a promising tool for sustainable water management strategies in the UNB and other similar catchments.

## Introduction

The hydrological processes have several interconnected elements, with runoff linking precipitation to water bodies. surface runoff refers to water movement across the soil surface and into nearby surface waters, such as streams, dams, lakes, or other reservoirs, without infiltrating into the ground. The variability of surface runoff is dependent on both temporal and spatial factors. Approximately 33% of the precipitation that reaches the field is converted into runoff, while the remaining 67% is either evaporated, infiltrated, or retained within the soil. According to the Central Water Commission (CWC), the total availability of water on the Earth is fixed, i.e., 1378.72 Mkm^3^ out of which 97% of water is in the oceans and 0.4 to 0.8% is trapped in the glaciers; only 2.2 to 2.6% of water is available as fresh water. However, the population of India is 17.85% of the world’s population, and it covers approximately 2% land area of the world. In contrast, it has only 4% of available water worldwide. Hence reliable streamflow prediction is significant for better water resources planning and management. The best model is one that describes a real-world system^1^. According to^2^, various hydrological models are classified as empirical, conceptual, physical-based, lumped, semi-distributed or distributed models. A runoff model comprises a series of equations that are utilized to estimate the runoff based on various parameters that define the characteristics of a watershed.

Several hydrological models are used to simulate rainfall-runoff processes, including SWAT, HEC-HMS, VIC, and WEAP, which are the usual models used by hydrological modellers across the world. For example, the potential of both surface water and groundwater to fulfil the demands of water for different users and the ecological reserve is assessed by implementing the WEAP model in a sub-basin of Steelpoort River, specifically called the Olifants River basin^3^. WEAP has also been considered to be a Decision Support System for water management planning. The study showed the importance of developing the Decision Support System and how the watershed system can use the developed DSS to enhance the flow dynamics in the basin management using the WEAP model^4^. The soil moisture method used in the WEAP model was employed to model the rainfall-runoff process while it modelled the hydrological system of the basin of Rio Conchos. The method showed that could be used to simulate the dynamics of the basin flow to assess the impacts of climate variation under several carbon dioxide emission scenarios^5^. WEAP model was also used to evaluate water supply and demand in the Langat watershed area. Due to the water balance in the catchment area, a new technique of inventory to match the changing availability of water due to pressure was developed. The result indicated that the analysis area was expected to face water shortage because of the increase in population and temperature rise above the current situation^6^. The use of WEAP will enhance synchronization between the hydrological input of the area and the water management system infrastructure. This system decides on the distribution of the available water resources to meet the varying water demands. Based on the findings, it can be inferred that constructing a hydroelectric dam on the Niger River would be advantageous as it would aid in regulating water flow and mitigate the issue of low water levels. Moreover, the dam’s construction will facilitate the acquisition of sufficient potable water for the prominent cities of Niamey and Tillabéry^7^.

In further study, WEAP model deployed and tested several unilateral and multilateral adaptation methods considering a socioeconomic and climatic change to analyse transboundary water resource management. WEAP has assessed agricultural and water policies in the Jordan River watershed to maintain rural livelihoods and safeguard freshwater resources^8^. WEAP model is used to forecast rainfall and temperature at a smaller spatial and temporal level to model the effect of climate change on water resources and provide results for water administrators and decision-makers. Climate change, he concluded, would have a direct impact on water sources and the demand for water in terms of uses such as urban and rural use could also be more disrupted. The capabilities of the WEAP21 water resource management model were assessed and tested when used in the Parameterization Simulation Optimisation system by the EGO method. The outcomes indicate that the WEAP21-PSO system has been successful, and EGO has the capacity and expertise to solve computationally difficult problems and yield the best solution according to^9^. WEAP was used for the quantitative predictions of the water supply and demand deficit in Chennai.” Furthermore, the impact of various potential long-term gradual water availability improvements on the water supply-demand failure was predicted using WEAP for the outcomes analysis. The data between 2009 and 2015 was used to analyze and validate the model, while the model calibration data were from 1991 to 2008. The results revealed that the system’s productivity increased with the construction of a second desalination plant, by harvesting water from the same reservoir and recycling wastewater^10^. The WEAP model for the Ur-River catchment in Maharashtra state was adapted, connected to IWRM, and employed as a simulation method to conduct the scenario method and assess the supply and demand of water. As findings, scenario methodology WEAP-MABIA could be performed efficiently, and many more suggested^11^. Water source access: testing the WEAP model by analyzing hydrological elements and calculating the Pradesh catchment’s water balance was essential for the accessibility of water resources, as is the case in the Chongwe River. The Actual Evapotranspiration is increasing by 0.03 Mm^3^/year, Potential Evapotranspiration decreasing by 0.24 Mm^3^/year, the annual stream flow is increasing by 0.13 Mm^3^/year, and precipitation p is decreasing by 0.12 Mm^3^/year. The WEAP process simulation results were statistically assessed by computing the coefficient of determination and the Nash-Sutcliffe function effectiveness coefficient. The process simulation results showed a good correlation with an R^2^ of 0.97 and an NSE of 0.64^12^.

Data-driven models are inspired by the information processing capabilities of a biological nervous system with available large data sets, such as the brain. Developing soft computing techniques like artificial neural networks (ANNs), support vector systems, and the adaptive neuro-fuzzy inference system to model stream-flows using the available data has also benefited from science and computer technology advances. It has been observed that among these soft computing tools, ANNs are increasingly in demand and used for various applications, such as streamflow projections and estimates of future hydropower generation, to comprehend links between future climate and crop yield. Although the ANN approach has been used in streamflow forecasting globally, the UNB has not received significant attention for this technique. Because the ANN models can describe linear and nonlinear systems without any assumptions, unlike most traditional statistical approaches, they are increasingly being employed in numerous science and engineering fields. ANNs were first developed in the 1940s by^13^. ANNs have been successfully utilized for the rainfall-runoff process^14^, to predict river flow in specific hydrologic issues^15^, for the characterization of soil pollution, and the prediction of water-quality parameters. Additionally, ANNs are used to anticipate rainfall, runoff, and evaporation, for river-flow time-series prediction and flood-disaster prediction. A multilayer feed-forward backpropagation method is employed in various hydrological applications. It typically consists of many interconnected nodes organized into an input layer, an output layer, and one or more hidden layers. The sigmoid function was chosen as the transfer function for the network. ANNs are a subclass of machine learning that has attracted a lot of attention in the context of estimating problems. Data processing devices called ANNs imitate the functions of the human brain^16^. Machine learning approaches’ popularity is growing due to their use, simplicity, and effectiveness. With little data and a complex process, machine-learning techniques are a great option^17^. In the last few decades, ANN models have been widely used in managing watersheds, water resources, and hydrology. Three layers make up the ANN: the input layer, the hidden layer, and the output layer. The interaction between the neurons in the subsequent levels determines how much communication there is^18^. ANN models are black-box models^19^. Applying ANNs in creating models results in trustworthy and versatile learning ability, making ANNs promising for forecasting^20^.

Although hydrological models, such as the WEAP and ANN, have been generally used in the modelling of the rainfall-runoff process, their limitation is associated with inefficient performance. Like, WEAP is used to simulate various complex water systems and account for hydrological processes. Furthermore, the use of the ANN model is due to its capability to generate data-driven models to establish nonlinear and complex patterns. However, there is limited existing literature on the development and application of the hybrid model that integrates the strengths of both the conceptual understanding of the hydrological process in WEAP and the literature-based learning abilities of the ANN model in the rainfall-runoff modelling. The use of physical hydrological models combined with machine learning techniques has emerged as a novel and effective approach to improving the accuracy and validity of rainfall-runoff simulations. This hybrid methodology combines the advantages of two different models: while physical hydrological models account for the understanding of underlying processes, machine learning is used to amend simulations by reconciling them with the observed data. Hybrid models have proven to be useful in alpine regions where both hydropower plants and glacial melt contribute to streamflow patterns^21^. combined the Soil and Water Assessment Tool (SWAT), with the Support Vector Machines (SVM) to model hydropeaking in the absence of the reservoir operation rules following the rigid schedules. The approach was very effective as the simulation error was decreased by tens of per cent. Another model combining the HBV hydrological model with a Bayesian neural network was employed by^22^ to improve monthly streamflow forecasting for the Yarkant River and account for the precipitation, snow, and glacier melting in the region. The HBNN method yielded very good results for the high flows^23^. used the approach to hybridize the WEAP, and the GR2M with the ANN. Their approach was particularly interesting as the output from the two models was further used as an input for the new ANN, which produced excellent results and brought NSE to 0.99 values from 0.64 to 0.88 of the initial models. The availability of several models that have been hybridized using various machine learning techniques and physical hydrological models is a further confirmation of their effectiveness. The LSTM-XAJ from^24^ was also proved to be extremely effective in flood forecasting using the multiple basins and the multi-step-ahead approach^25^. provided an example of the further development of a nested hybrid model that accounted both for the physical processes and for the non-linear patterns in conventional data. Recent advances in the SWAT, LSTM, and RF model by^26^ proved to be more effective and valid than the conventional models. Comparative studies of machine learning approaches and conventional hydrological models have also indicated that hybrid models should be developed. Further^27^, indicated that LSTM surpasses the machine learning models and also the SWAT models in the glaciated region of the Tianshan Mountains. Moreover, other studies by^28–30^ have demonstrated that AI and machine learning models can bring similar or even better results than physical process-driven models in case of some limitations of the data. All of the above findings indicate that, rather than replacing, the machine learning hydrological models could be combined with the process-driven ones.

The hybrid models are also promising, as they could be employed not only in streamflow forecasting but also in parameter regionalization and uncertainty quantification^31^. For instance^32^, have demonstrated that SWAT model performance could be enhanced using machine learning models and, in particular, the approach of so parameter estimation through machine learning leading to significant improvements in the model calibration. Another method developed by^33^ used decision tree algorithms and the limits of acceptability (LOA), to narrow the parameter range for more accurate and less complex hydrological predictions. The emerging models go even further –^34^ have introduced a model that hybridized the conventional process-based models with CNN and LSTM^35^. went even further by introducing super ensemble deep learning models that are even more effective than conventional conceptual hydrological models. The effectiveness of hybrid models in complex regions and data-constrained regions with complex hydrological process were proven from different approaches by^36–38^.

Additional to it, recent studies have emphasized the importance of spatial pattern analysis and hydrological modeling in understanding precipitation variability^39,40^. employed data-driven models and clustering techniques to simulate water levels and characterize spatiotemporal properties of precipitation, respectively^41^. analyzed trends and variability in monthly rainfall, while^42^ investigated the synoptic conditions leading to floods and the relationship between ENSO and streamflow. These studies highlight the significance of understanding spatial and temporal patterns of precipitation and the meteorological aspects of flooding for effective water resource management. Similarly, few more studies have highlighted the growing importance of machine learning techniques, particularly hybrid and ensemble approaches, in hydrological modeling. These methods have been successfully applied to various problems, such as suspended sediment load prediction^43^, groundwater level prediction^44^, rainfall-runoff modeling^44,45^, daily flow discharge prediction^46^, and precipitation prediction^47^. These studies underscore the significance of exploring novel methodologies to enhance the accuracy and robustness of hydrological predictions, aligning with the objectives of the present study.

Despite the extensive use of hydrological models like WEAP and ANN in rainfall-runoff modeling, their performance is often limited in data-scarce catchments^48,49^. While some studies have explored combining physical and data-driven models to improve streamflow predictions^50^, the potential of such hybrid approaches in data-limited regions remains largely unexplored.

To address this gap, we propose a novel hybrid technique, ANN_Hybrid_, that integrates simulated flow from both a physically-based distributed hydrological model (WEAP) and a data-driven model (ANN) to enhance streamflow prediction accuracy in data-scarce catchments. The effectiveness of the proposed approach is evaluated against standalone WEAP and ANN models in the UNB, which lacks long-term hydrological observations. The main contributions of this study are: (1) demonstrating the effectiveness of combining a physically-based hydrological model (WEAP) with machine learning techniques (ANN) to enhance streamflow prediction accuracy in data-scarce catchments; (2) proposing a novel hybrid technique, ANN_Hybrid_, that integrates simulated flow from both physical and data-driven models to further improve the performance of streamflow simulation; and (3) evaluating the performance of the proposed hybrid approach against individual WEAP and ANN models in the UNB. The findings provide valuable insights for developing accurate and efficient streamflow prediction models in data-limited regions, which is crucial for effective water resource management and planning.

## Study area

**Figure. 1:**
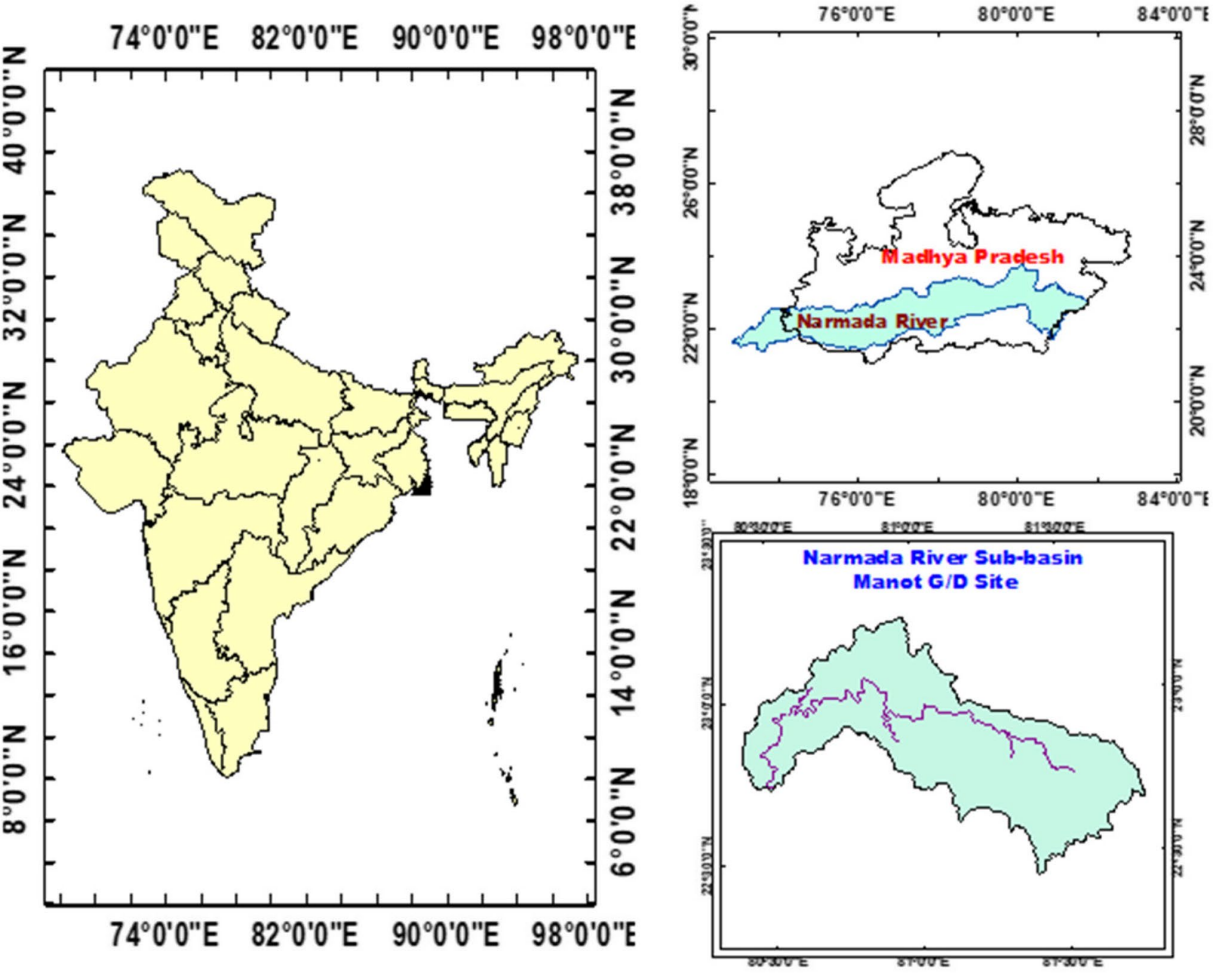
Narmada River sub-basin.

The study area of this study is UNB. Narmada River is a west-flowing river in Central India and the 5th largest river in the sub-continent of India. It is the third-longest river which flows totally within India, after Krishna and Godavari. It flows through the rift valley between the Satpura and Vindhyan ranges. The river originated from the Amarkantak Plateau of Maikala, located in the Shahdol district of Madhya Pradesh. The source coordinates are 22°40’ North latitude and 81°45’ East longitude, with an altitude of 1057 m above the mean sea level. The map depicted in Fig. 1 displays the study area spanning from the source of the Narmada River to the Manot G/D site, which includes an area of approximately 4765 square kilometres.

The UNB was chosen for this research due to its importance in the region and the need for accurate streamflow predictions to support water resource management. The sub-basin is characterized by diverse hydrological conditions and land use patterns, making it an ideal site for testing the performance of the proposed hybrid modeling technique that integrates the physical WEAP model with data-driven approaches like ANN and a categorization approach. Furthermore, the UNB serves as a representative case study for demonstrating the applicability and effectiveness of the proposed ANN_Hybrid_ modeling technique. The findings from this research can be extended to other river basins facing similar challenges, highlighting the potential of hybrid modeling approaches in enhancing rainfall-runoff predictions and supporting sustainable water resource management^51^.

The study area is divided into five parts according to the land type shown in Table 1. A significant portion of the basin’s precipitation occurs during the southwest monsoon and accounts for about 85–95% of the total rainfall. The relative humidity in the basin varies between 92% and 27% in the morning and between 88% and 15% in the evening, depending on the season. This basin consists mainly of black soil. Black soils have high water-holding capacity, and the organic matter is generally less than 5% in black soils.

**Table 1:** Classification of the study area based on land use.

Land Type	Agriculture	Forest	Grassland	Urban	Open water
Area (Hectares)	212,621	204,852	58,568	43	416

## Methods

**Figure. 2:**
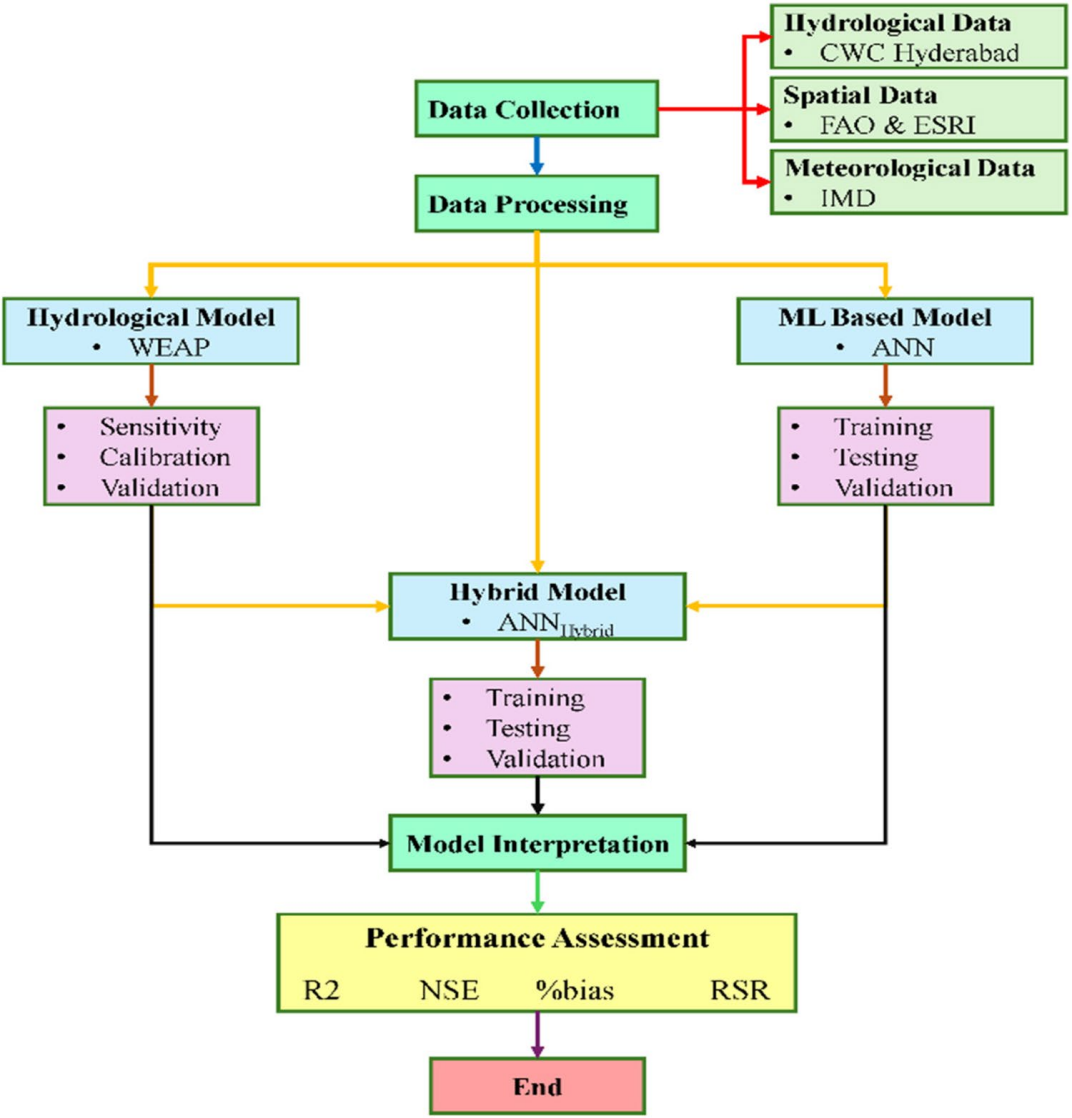
Flowchart for methodology.

Figure 2 shows the detailed flowchart of methodology used for present study.

### Data used

The model’s primary input data requirements include meteorological and discharge data for catchment parameter definition, initial condition determination, and model calibration and validation. Meteorological data include precipitation, wind speed, temperature, and humidity. The data used in this study were obtained from reliable and authorized sources, ensuring their integrity and quality. The meteorological data, including precipitation, temperature, relative humidity, and wind speed, were collected from the Indian Meteorological Department (IMD). The observed streamflow data used for model calibration and validation were obtained from the Central Water Commission (CWC) of India. All data were used solely for research purposes, and no personal or sensitive information was involved in the study. The investigation has complied with the data usage policies and guidelines set by the respective organizations and have duly acknowledged the data sources.

#### Rainfall

**Figure. 3:**
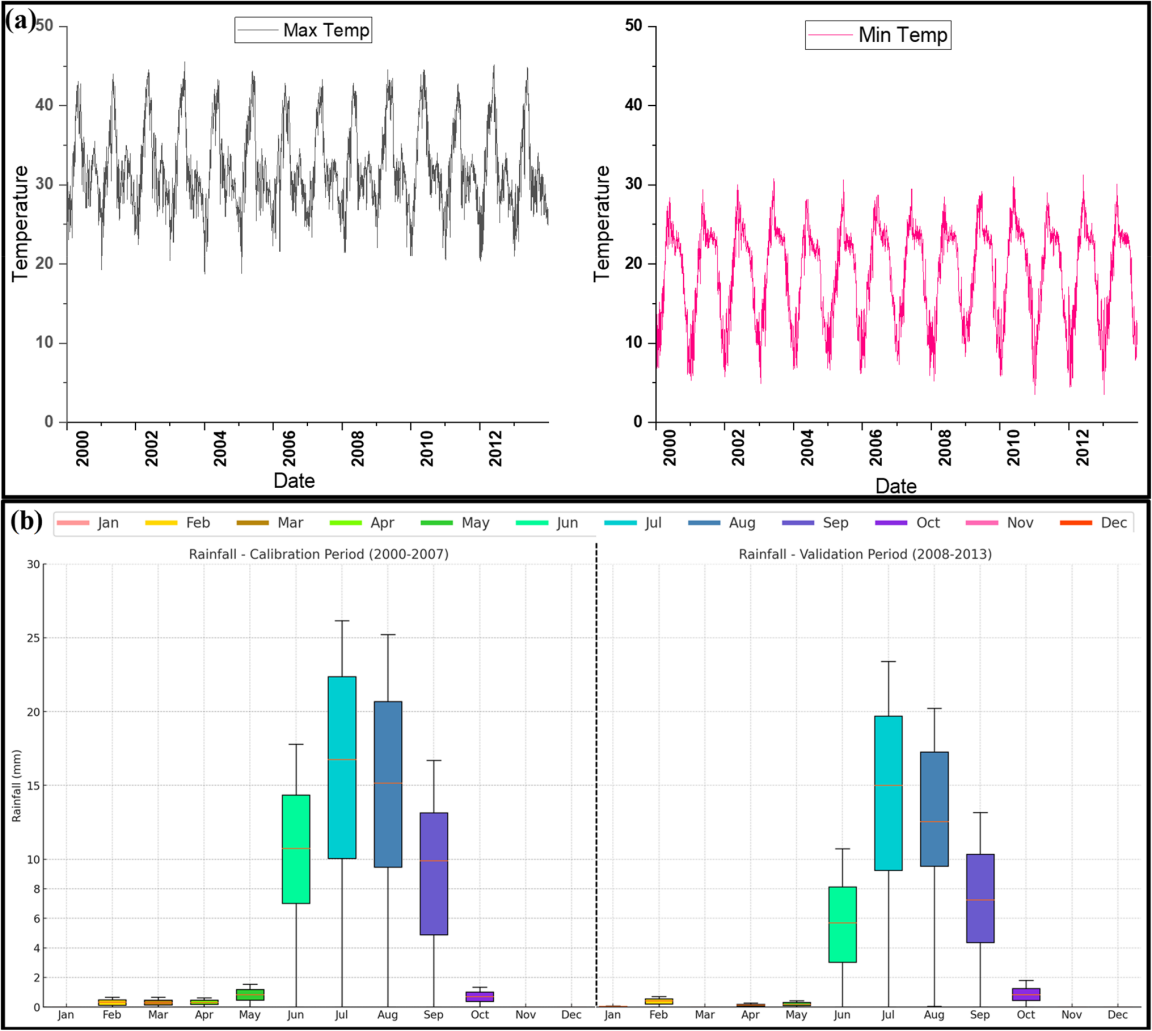
(**a**) Daily maximum and minimum temperature. (**b**) Box plot of monthly rainfall distribution in calibration and validation time period.

The daily gridded precipitation at a resolution of 0.25°x0.25° (IMD) has been used in the study area. The Thiessen Polygon method was used to compute the daily aerial average rainfall over the study area. The daily rainfall data of 8 grid points spread over the catchment for calculating the average daily rainfall. The location of all rain grids over the catchment area and the weightage for those grids are shown in Table 2. Figure 3(b) show the distribution of observed monthly rainfall in UNB for calibration and validation period.

#### Wind speed & humidity

The humidity and wind speed characteristics of the UNB play an important role in determining local climate and influence various hydrological and ecological processes. Seasonal fluctuations are observed in the wind speed, with higher velocities during summer and comparatively slow winds during winter. The humidity levels also exhibit seasonal fluctuations, with higher values during the monsoon season and lower values during summer. These variables significantly influence the rates of evaporation, water availability, and the survival of flora and fauna. They are crucial factors when implementing hydrological studies in any river basin or sub-basin.

#### Temperature (maximum & minimum)

There are noticeable seasonal temperature variations in the UNB. Maximum temperatures during the summer vary from 18 °C to 45 °C, while minimum temperature falls between 5 °C and 30 °C. Figure 3(a) shows the variations of daily maximum and minimum recorded temperature (IMD) for UNB. The hydrological cycle may be significantly impacted by these high temperatures, including increased evaporation rates and water scarcity. Also, the ecological processes impacted by the low temperature include plant growth, soil moisture levels, and aquatic organisms’ patterns of survival and reproduction.

**Table 2:** Details of the influencing rainfall grides.

Influencing Rain Grids	Latitude Longitude	Weights	Area (sq. km)	Location
01	22.75 N; 80.5E	0.052	251	
02	22.75 N; 80.75E	0.004	022	
03	22.75 N; 81E	0.074	357	
04	23 N; 80.5E	0.060	289	
05	23 N; 80.75E	0.160	767	
06	23 N; 81E	0.188	896	
07	22.75 N; 81.25	0.188	896	
08	22.75 N; 81.5 E	0.264	1314	

#### Runoff

**Figure. 4:**
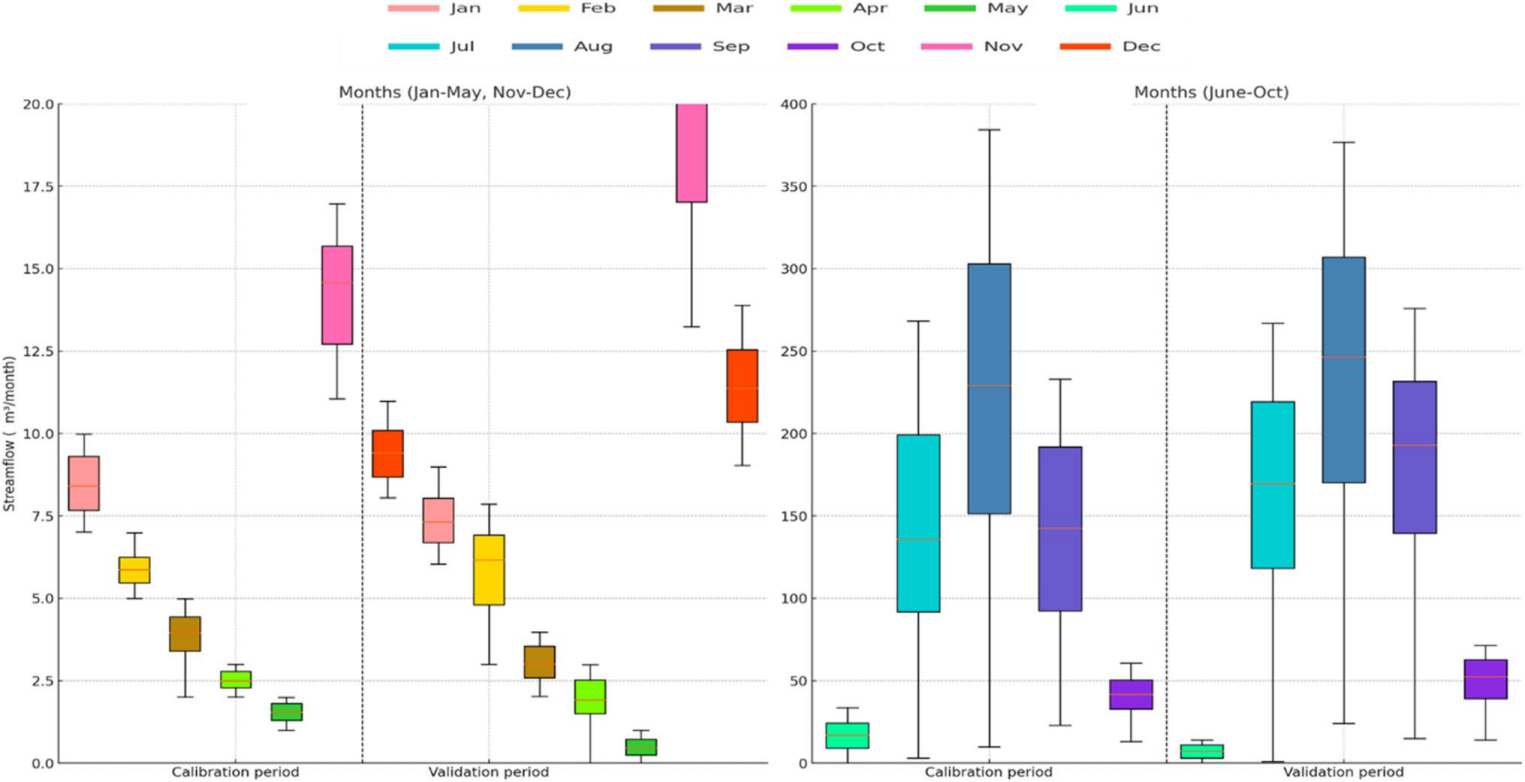
Box plot of monthly flow distribution in calibration and validation period.

The runoff at the stream gauge station located at Manot (CWC) has been used to calibrate/train and validate/test the WEAP/ANN model respectively. The daily runoff from January 2000 to December 2013 was available and used in the present study. The runoff coefficient for the calibration and validation period is given in Table 3, and Fig. 4 shows monthly flow distribution in monsoon and non-monsoon months for calibration and validation period.

**Table 3:** Details of the rainfall and runoff data for the calibration and validation period.

Year	Rainfall (mm)	Runoff (mm)	Runoff Coefficient
2000	921.81	440.24	0.48
2001	1377.14	801.92	0.58
2002	969.10	430.41	0.44
2003	1476.60	967.87	0.66
2004	1236.98	765.78	0.62
2005	1585.37	1016.73	0.64
2006	990.24	573.90	0.57
2000-06	8557.24	4996.85	0.57
2008	1125.12	517.9	0.46
2009	831.43	278.97	0.34
2010	1090.07	434.54	0.40
2011	1490.18	864.83	0.58
2012	1083.84	507.51	0.47
2013	1421.73	635.33	0.45
2008-13	7042.37	3239.08	0.45

### Water Evaluation and Planning (WEAP) model

The WEAP model was developed by the Stockholm Environment Institute (SEI). The system is a critical simulation tool with multiple reservoirs and purposes, which selects the optimal water distribution approach by prioritizing supply and demand aspects. The programme provides a user-friendly and adaptable method for planning and policy creation. Using an integrated method, natural inflows, and artificial components, such as water reservoirs and groundwater pumps, are both simulated by WEAP. This provides a realistic perspective on the issues that are addressed in managing current and future use of water resources. This model enables the prediction of the entire system’s effects and facilitates the analysis of diverse water development and management options (SEI, 2007). WEAP employs a simple collection of model devices and procedures to examine water managers’ various problems and uncertainties, such as the environment, watershed conditions, anticipated demand, ecosystem needs, regulatory environment, functional priorities, and infrastructure^52^.

There are nine parameters of WEAP, such as Soil-Water-Capacity (SWC); Root-Zone-Conductivity (RZC); Runoff-Resistance-Factor (RRF); Preferred-Flow-Direction (PFD); Deep-Water-Capacity (DWC); Deep-Conductivity (DC); Crop Coefficient (Kc); Initial-$\:{\text{Z}}_{1}$; and Initial-$\:{\text{Z}}_{2}$. The SWC is the adequate water-holding capacity of the upper soil layer given in mm. The SWC varies according to the type of land class. Moreover, RZC is the saturated hydraulic conductivity of the topsoil layer and varies according to the different land classes. Furthermore, RRF controls surface runoff and the increase in the RRF values will increase the resistance to flow and reduce runoff. In addition, PFD is a parameter that is used to partition the flow between the topsoil layer (surface runoff) and flow to the lower soil layer (baseflow) and PFD varies depending on the soil type in the catchment. Moreover, DWC depicts the adequate water-holding capacity of the lower soil layer and is given in mm which is uniform across all types of land classes. DC is the saturated hydraulic conductivity of the bottom soil layer and does not vary according to the land classes that are responsible for controlling the transmission of baseflow. Along with all the parameters, Initial-$\:{\text{Z}}_{1}$ relative storage and Initial-$\:{\text{Z}}_{2}$ relative storage are expressed as a percentage of the total adequate storage of the topsoil water capacity at the beginning and the total adequate storage of the bottom layer of soil respectively.

### Artificial-Neural-Network (ANN) model

ANN has been successfully applied in a wide range of domains such as the classification of data; data mining; speech recognition; time series analysis etc. The ANN models are effective forecasting tools for the relationship between runoff variables and rainfall. The findings will aid in decision-making on managing and planning water resources. Additionally, they help managers and urban planners take the required steps to prepare for negative projections. As a result, it helps in preventing risks to human health and the environment that floods are expected to cause.

ANN is a set of connected inputs and output layers in which each connection has associated some weights. The mathematical computing program MATLAB was used to model the relationship between rainfall and runoff. The neural network was modelled using one hidden layer along with the input and output layers. Meteorological data such as daily rainfall, wind speed, temperature, humidity and one day before runoff were provided as input data, while hydrological data and observed runoff were fed as target data. During the learning phase, ANN learns by adjusting the weights to be able to predict the correct output according to input data. One of the three training methods in ANN, Levenberg-Marquardt (LM) was used to train the created ANN network. The training phase of ANN consists of three steps i.e., Initialize the weights for different inputs; propagate the input forward; and backpropagate the error. In the study sigmoid function was used as an activation function/transfer function which given as

1 $$\mathrm f\left(\mathrm{Sum}\right)=\frac1{1+\mathrm e^{-\mathrm{Sum}}}$$

2 $$\mathrm{Sum}=\sum\:_{\mathrm i=1}^{\mathrm n}{\mathrm I}_{\mathrm i}{\mathrm W}_{\mathrm i}+\mathrm b$$

where, I_i_ = input variable; Wi = Weights for connection of input layer to hidden layer; b = bias.

**Figure. 5:**
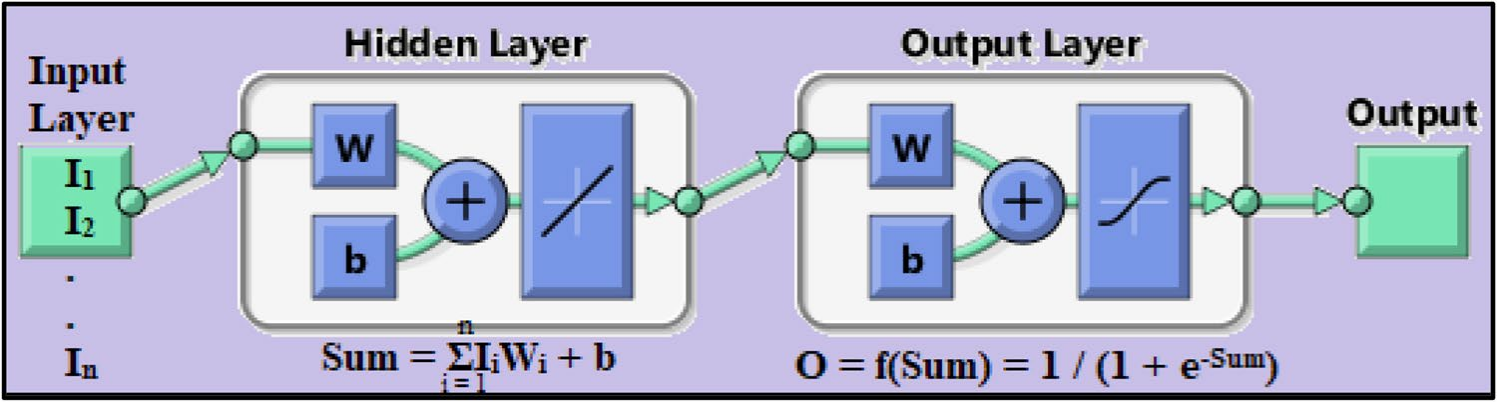
ANN network.

The ANN network used in the present study is shown in Fig. 5 and, it consists of 4 data in the input layer, namely (rainfall, temperature, one day before rainfall, and one day before runoff). The hidden layer consists of 5 sets of hidden neurons, i.e., 4, 5, 6, 7, and 8. The output layer comprises observed runoff.

The use of the ANN model in this study is justified by its proven effectiveness in capturing complex, nonlinear relationships between hydrological variables and streamflow^53,54^. ANNs have been widely applied in rainfall-runoff modeling due to their ability to learn from data and adapt to varying hydrological conditions^55^. Although ANNs have been used in numerous studies, their application in hybrid models, particularly in combination with physically-based models like WEAP, has been less explored. The novelty of this study lies in the development of the ANN_Hybrid_ model, which leverages the strengths of both the physically-based WEAP model and the data-driven ANN model to improve streamflow predictions in data-scarce regions.

The choice of the ANN model is further supported by its flexibility in incorporating various input variables, its ability to handle missing or incomplete data, and its computational efficiency compared to other ML techniques^56^. Additionally, the use of a well-established ML model allows for a more focused evaluation of the proposed hybrid approach, as the emphasis is on the integration of the physical and data-driven models rather than on the development of a new ML technique. By demonstrating the effectiveness of the ANN_Hybrid_ model in the UNB, this study highlights the potential of combining physically-based and data-driven models for improved streamflow predictions in data-limited regions. The findings provide a foundation for future research on hybrid modeling approaches and their application in water resource management.

### Performance evaluation criteria

The performance evaluation is done based on statistical indices of performance evaluation and comparing the observed and simulated runoff.

#### Coefficient of determination (R^2^)

R^2^ have been widely used for the performance evaluation of models. The value of R^2^ ranges from 0 to 1, where a higher value of R^2^ indicates less error variance, and typically values greater than 0.5 are considered acceptable for any model^25,27,38,57^.

3 $$\mathrm R^2=\left[\frac{\mathrm n\left(\sum\mathrm Y^{\mathrm{obs}}\mathrm Y^{\mathrm{sim}}\right)-\left(\sum\mathrm Y^{\mathrm{obs}}\right)\left(\sum\mathrm Y^{\mathrm{sim}}\right)}{\sqrt{\left\{\mathrm n\sum\left(\mathrm Y^{\mathrm{obs}}\right)^2-\left(\sum\mathrm Y^{\mathrm{obs}}\right)^2\right\}\left\{\mathrm n\sum\left(\mathrm Y^{\mathrm{sim}}\right)^2-\left(\sum\mathrm Y^{\mathrm{sim}}\right)^2\right\}}}\right]^2$$

Y^obs^ is the observed runoff, Y^sim^ is the simulated runoff, and n is the total number of observations in the evaluated time series.

#### Nash Sutcliffe Efficiency (NSE)

NSE indicates how well the plot of observed data versus simulated data fits the 1:1 line. A higher value shows a better relationship between observed and simulated values^21,24,27,38,57^.

4 $$\mathrm{NSE}=\left[\frac{\sum_{\mathrm i=1}^{\mathrm n}\left(\mathrm Y{}^{\mathrm{obs}}-\mathrm Y{}^{\mathrm{sim}}\right)^2}{\sum_{\mathrm i=1}^{\mathrm n}\left(\mathrm Y{}^{\mathrm{obs}}-\mathrm Y_{\mathrm{mean}}^{\mathrm{obs}}\right)^2}\right]$$

where *Y*^*mean*^ is the mean of observed runoff.

#### Percentage Bias (%bias)

%bias refers to the percentage deviation of evaluated data. The measure of %bias determines the average inclination of the simulated data to either exceed or fall short of their observed. Positive values signify underestimation by the model, while negative values indicate overestimation^27,38,58^.

5 $$\%\mathrm{bias}=\left[\frac{\sum_{\mathrm i=1}^{\mathrm n}\left(\mathrm Y_{\mathrm i}^{\mathrm{obs}}-\:\mathrm Y_{\mathrm i}^{\mathrm{sim}}\right)\ast\left(100\right)}{\sum\:_{\mathrm i=1}^{\mathrm n}{(\mathrm Y}_{\mathrm i}^{\mathrm{obs}})}\right]$$

#### RMSE-observations standard deviation ratio (RSR)

RSR ranges from a high positive value to the ideal value of zero. The performance of the model simulation increases with decreasing RSR^21^. RSR is calculated as

6 $$\mathrm{RSR}=\frac{\mathrm{RMSE}}{{\mathrm{STDEV}}_{\mathrm{obs}}}=\frac{\left[\sqrt{\sum_{\mathrm i=1}^{\mathrm n}\left(\mathrm Y_{\mathrm i}^{\mathrm{obs}}-\mathrm Y_{\mathrm i}^{\mathrm{sim}}\right)^2}\right]}{\left[\sqrt{\sum_{\mathrm i=1}^{\mathrm n}\left(\mathrm Y_{\mathrm i}^{\mathrm{obs}}-\mathrm Y^{\mathrm{mean}}\right)^2}\right]}$$

### Ethical approval

This material has not been published in whole or in part elsewhere; The manuscript is not currently being considered for publication in another journal; All authors have been personally, and actively involved in substantive work leading to the manuscript and will hold themselves jointly and individually responsible for its content.

## Results

### Sensitivity analysis of WEAP

**Figure. 6:**
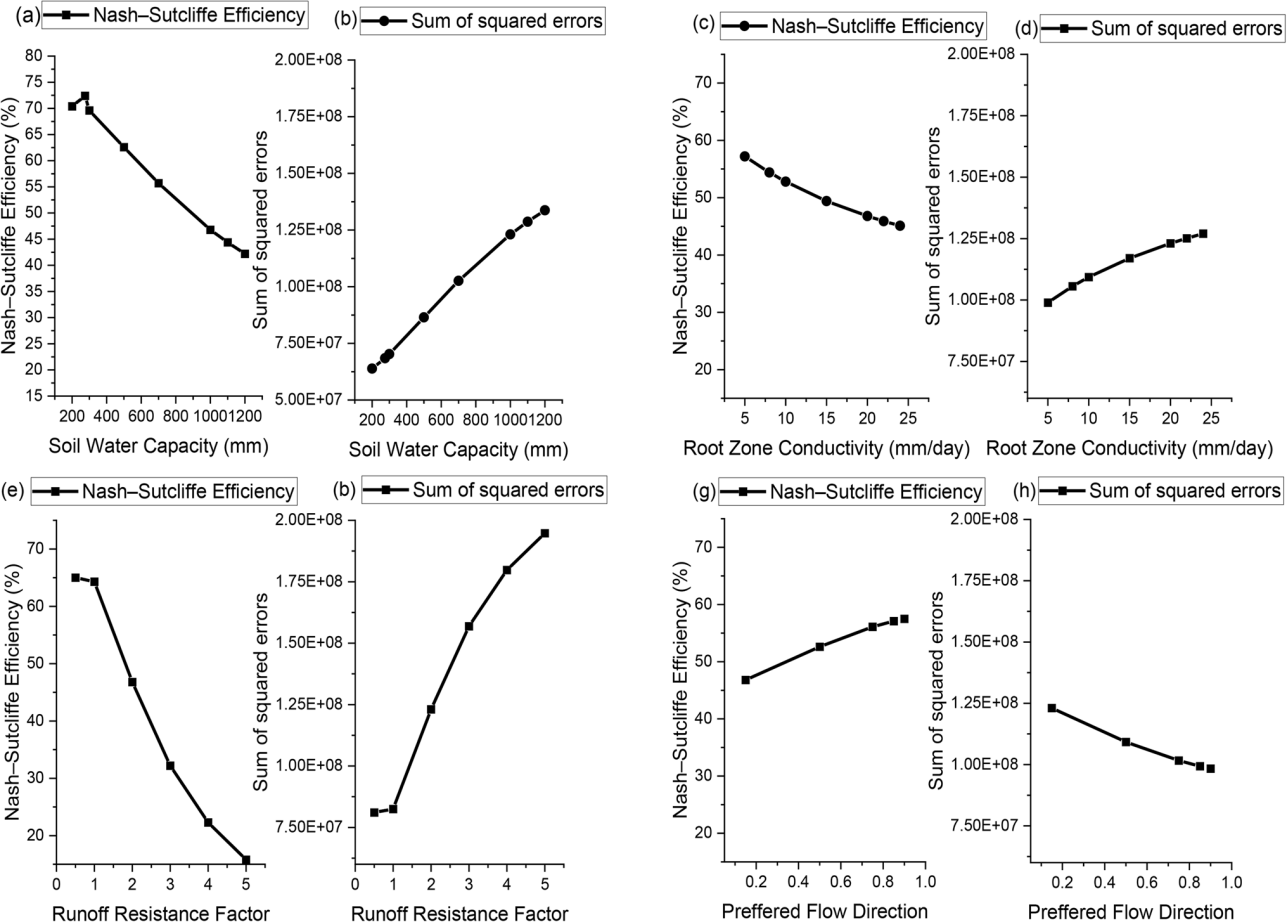
Sensitivity analysis of SWC (**a** & **b**); RZC (**c** & **d**); RRF (**e** & **f**); and PFD (**g** & **h**) based on NSE and SSE.

In the present study runoff data was set as output and the minimum & maximum temperature, rainfall, wind speed and relative humidity was set as input since they are the main causes which effects the runoff. Soil Moisture method in WEAP model was used for the prediction of streamflow. Where, Soil Moisture Method works in such a way that it automatically utilizes 9 key parameters related to soil characteristics, hydrological behavior, and initial conditions to simulate streamflow and other hydrological processes^59,60^. These parameters include Crop Coefficient, Soil Water Capacity, Deep Water Capacity, Runoff Resistance Factor, Conductivity of Root Zone, Conductivity of Deep Zone, Preferred Flow Direction, Initial Z1 and Initial Z2^59,61^. The sensitivity analysis of model was done by adjusting these parameters to identify the most sensitive parameters that have the greatest impact on model outputs^51^. Figure 6 shows the result of the sensitivity analysis of four sensitive parameters, i.e., SWC; RZC; RRF; and PFD based on NSE and SSE.

### WEAP model calibration

**Figure. 7:**
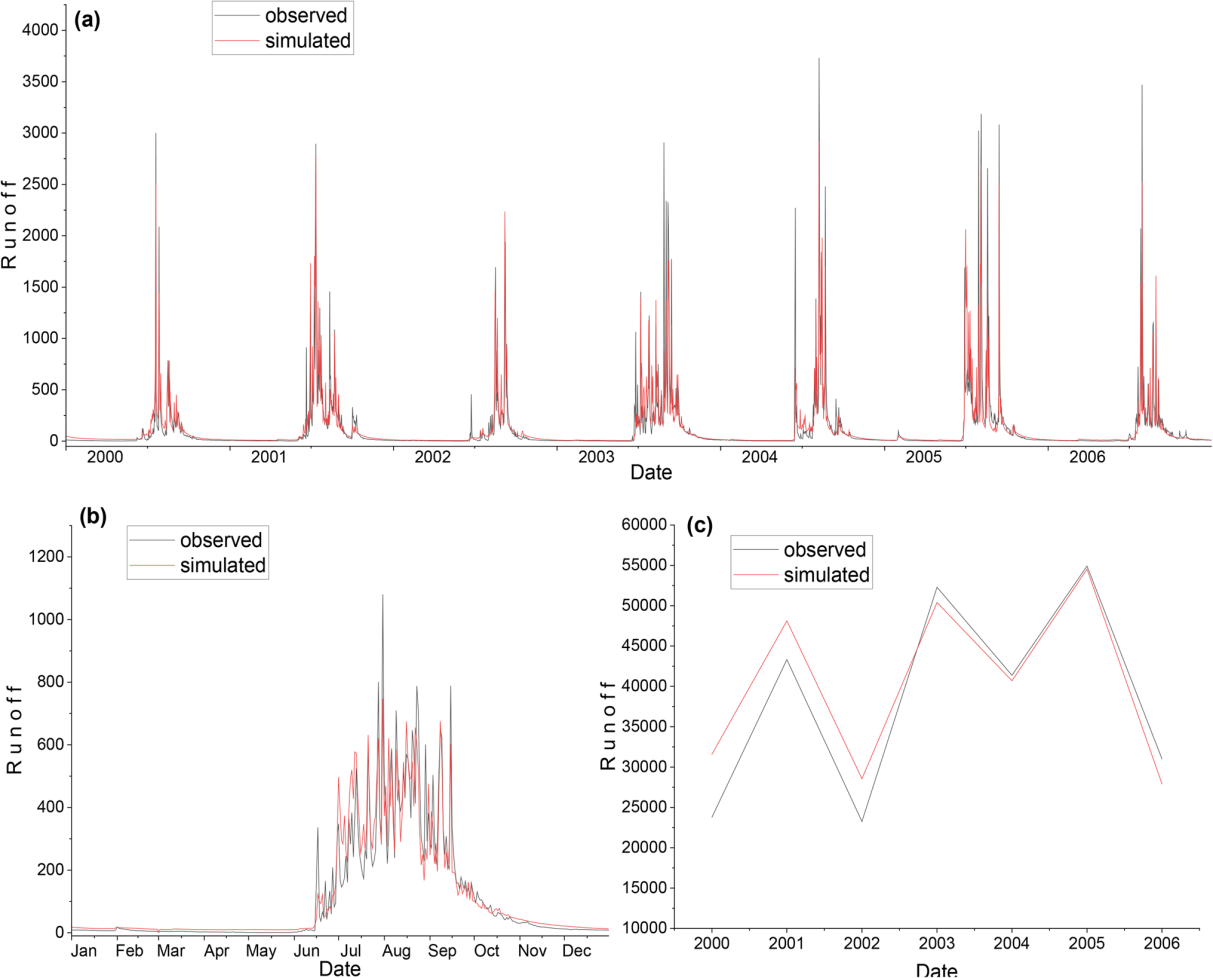
Comparison of observed and simulated runoff during the calibration using WEAP. (**a**) daily; (**b**) average daily; & (**c**) annual.

The selection of appropriate parameter values is crucial for the accurate simulation of hydrological processes in the WEAP model. In this study, a sensitivity analysis was performed to identify the most influential parameters affecting the model’s performance^51^. The sensitive parameters identified were subjected to a manual calibration process, where their values were adjusted within physically plausible ranges to minimize the discrepancy between observed and simulated streamflow^12,62^. The calibration was performed using a trial-and-error approach, to maximize the Nash-Sutcliffe Efficiency (NSE) and minimize the Sum of Squared Errors (SSE)^63^. The final calibrated parameter values were selected based on their ability to provide the best match between observed and simulated streamflow, as evaluated by the performance metrics discussed in the “Performance Evaluation Criteria” section. This systematic approach to parameter sensitivity analysis and calibration ensures that the WEAP model is optimally configured to represent the hydrological characteristics of the UNB, thereby enhancing the reliability and accuracy of the simulated results. The model was calibrated for seven years, from 2000 to 2006. Table 4 shows the final model parameters values of the calibrated model, and Table 5 displays the performance of the model in the calibration period and performance indicates good values, i.e., NSE = 75%, % Bias = -4.4, RSR = 0.48 and R^2^ = 0.754 which depicts good resemblance between observed and simulated runoff. Figure 7(a), (b), and (c) shows the comparison of the daily, average daily and annually observed and simulated runoff, respectively during the calibration period.

**Table 4:** Calibrated model parameters values.

Parameter	Parameter value
SWC	275 mm
DWC	400 mm
RZC	5 mm/day
DC	2 mm/day
RRF	4
PFD	0.9*
Initial Z_1_	30%
Initial Z_2_	50%

**Table 5:** Performance evaluation of the model during calibration.

Year	NSE (%)	*R* ^2^	%Bias	*R*.S.*R*.
2000	85.4	0.86	-32.8	0.38
2001	74.6	0.79	-11.0	0.50
2002	76.7	0.81	+ 03.5	0.48
2003	69.1	0.69	+ 01.6	0.55
2004	82.4	0.83	+ 00.7	0.41
2005	67.1	0.68	+ 09.9	0.57
2006	81.4	0.83	-04.4	0.43
2000–2006	75%	0.754	-4.4	0.48

### WEAP model validation

**Figure. 8:**
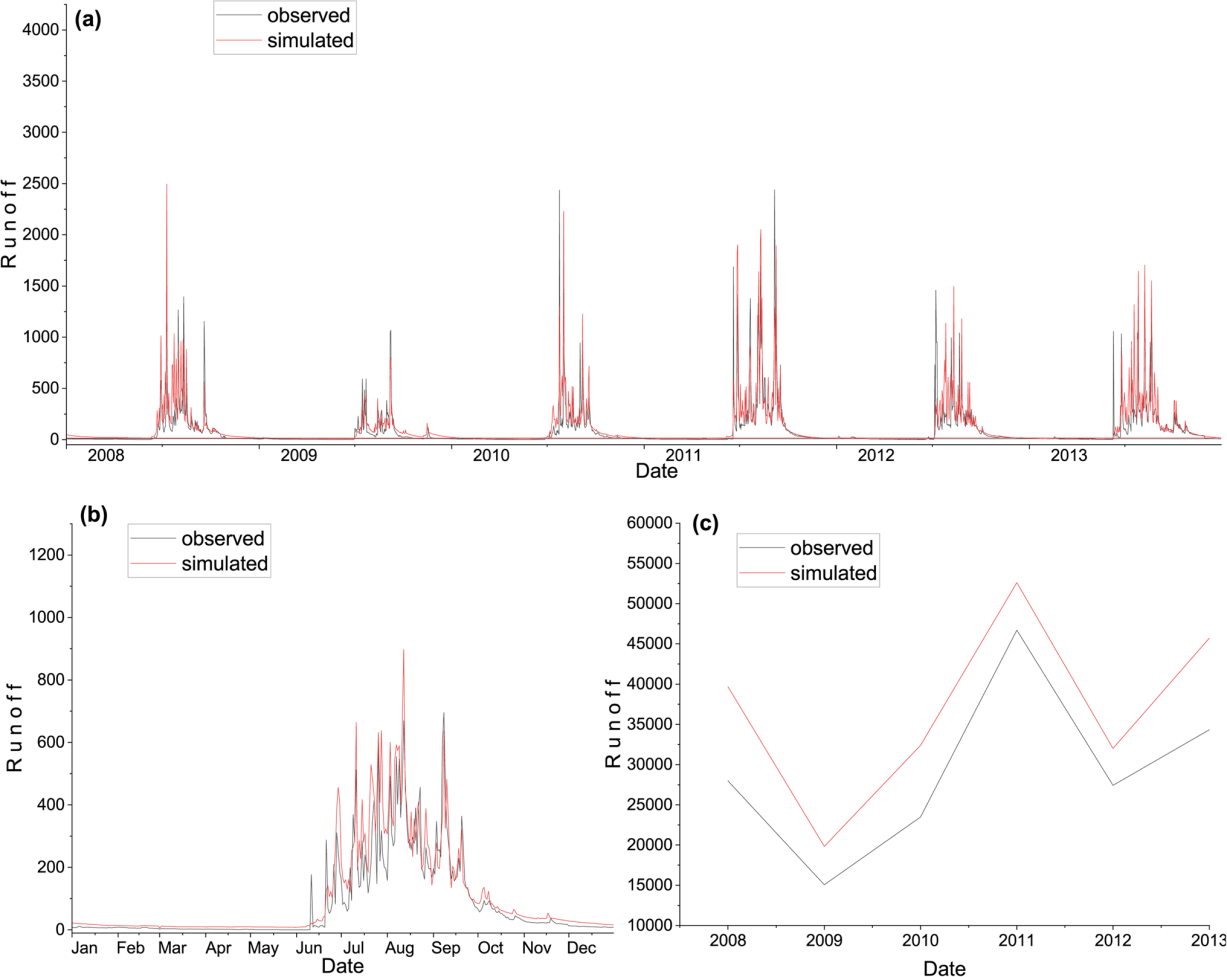
Comparison of observed and simulated runoff during the validation using WEAP. (**a**) daily; (**b**) average daily; & (**c**) annual.

The model validation has been carried out to test the ability of the model to simulate the runoff with the independent rainfall data and for periods other than those used in the calibration process. The validation was carried out for six years, from 2008 to 2013. The calibrated model was run with the independent rainfall data from 2008 to 2013 using the calibrated model parameters to simulate runoff. The output of the simulations during the validation period was evaluated using the same statistical indices used to evaluate the model performance in Table 6. Figure 8(a), (b), and (c) shows the comparison of the daily, average daily annually observed and simulated runoff, respectively, during the validation period.

**Table 6:** Performance evaluation of the model during validation.

Year	NSE (%)	*R* ^2^	%Bias	*R*.S.*R*.
2008	41.2	0.62	+ 41.8	0.63
2009	71.7	0.74	-31.6	0.53
2010	70.3	0.73	-37.9	0.54
2011	68.6	0.73	-12.6	0.56
2012	38.3	0.49	-16.7	0.78
2013	48.6	0.69	-33.2	0.71
2008–2013	59.2	0.67	-27.0	0.64

As the result presented, during the validation of the WEAP model, the runoff was overestimated considerably because the runoff coefficient was significantly less during the validation period as compared to the calibration period, this means that the model parameters were calibrated to generate higher runoff due to this, it generated higher runoff during the validation period.

### ANN model

Various combinations of input data sets and several hidden neurons in the hidden layer were used to develop different ANN models to perform rainfall-runoff modelling and simultaneously determine the values of performance evaluation metrics, such as R^2^, NSE, %bias, and RSR for each ANN. A neural network with only one hidden layer having (2n + 1) hidden neurons, where ‘n’ is the number of input nodes, can represent any continuous function. The correct number of neurons to use in the hidden layers can be determined using different thumb rules, such as – (1) The number of nodes in the hidden layer should be between the size of the input and output layers. (2) Number of nodes in the hidden layer should be 2/3 of the total nodes in the input & output layer. (3) Number of nodes in the hidden layer should be less than two times of nodes in the input layer. These three guidelines give an idea about the number of hidden neurons in a hidden layer at the start. Still, it will ultimately come from trial and error when selecting an architecture for your neural network.

**Figure. 9:**
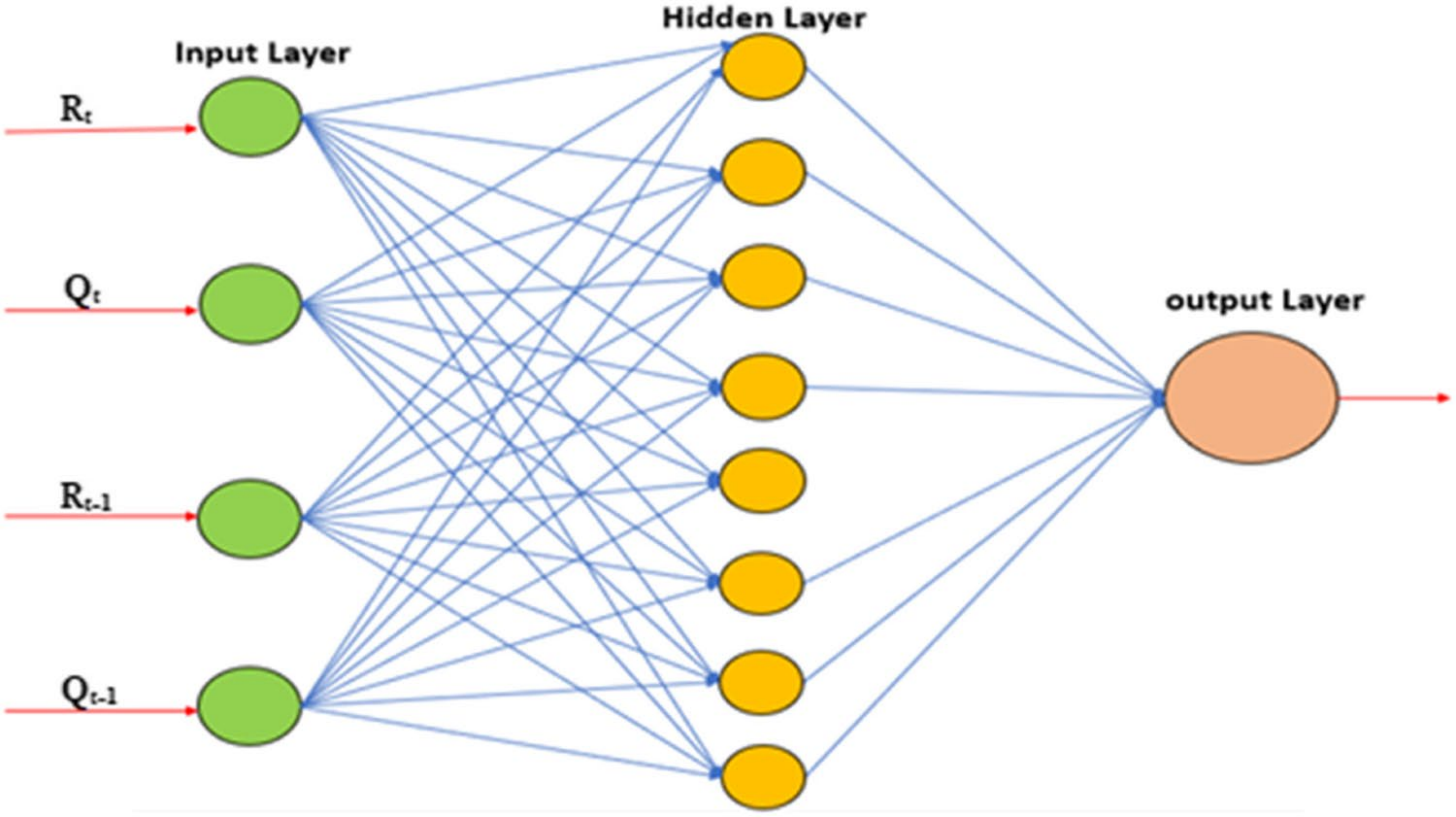
Feed forward back propagation ANN model (4-8-1).

**Figure. 10:**
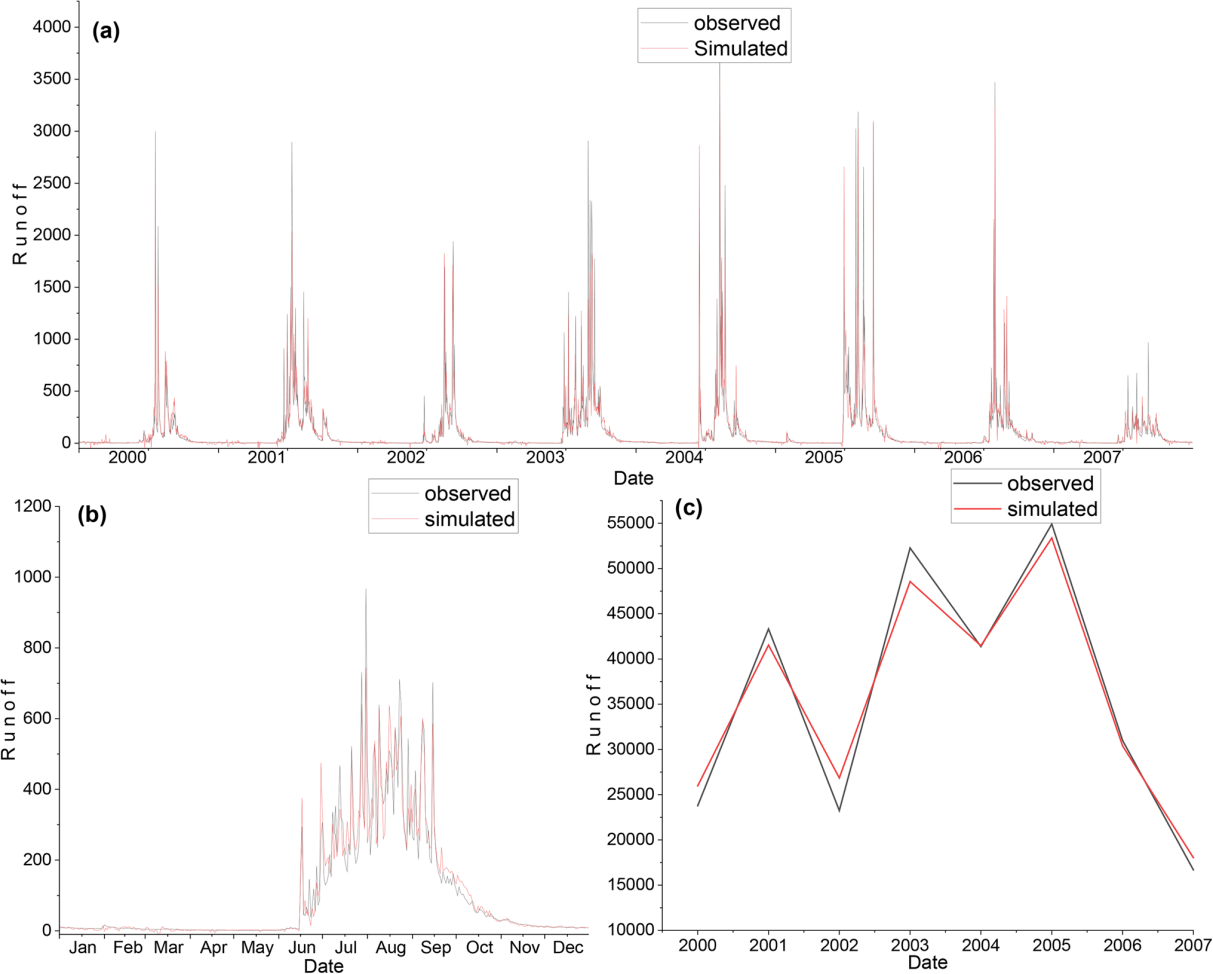
Comparison of observed and simulated runoff using the ANN model during the training period. (**a**) daily; (**b**) average daily; (**c**) annual.

Further, many neural networks were created for different input data sets and numbers of neurons in the hidden layer and check the performance of each network, shown in Table 7. Among all input data sets, the combination of R_t_; T_t_; Q_t−1_; & R_t−1_ shows the best result. For this data set model with different numbers of hidden neurons developed i.e., 4-4-1, 4-5-1, 4-6-1, 4-7-1, & 4-8-1, out of these networks structure of 4-8-1 ANN shown in Fig. 9, gives the best simulation and shows good correlation between observed and simulated runoff. However, when the input data combination changed by adding other variables, the results showed negligible variation. The output of the simulations through ANN was evaluated using the same statistical indices used to evaluate the model performance for WEAP, shown in Table 8 for the training and testing periods respectively. Figures 10(a), (b), & (c) and 11(a), (b), & (c) shows the comparison of the daily, average daily, annually observed, and simulated runoff, during the training and testing period respectively.

**Table 7:** Comparisons of ANN Models with different input data sets.

No. of Input parameters	Name of Input Parameters	ANN Model	Calibration				Validation			
			NSE %	*R* ^2^	%Bias	RSR	NSE %	*R* ^2^	%Bias	RSR
03	R_t_; T_t_; Q_t−1_	3-5-1	78.4	0.89	0.97	0.45	75.2	0.91	-5.65	0.49
04	R_t_; T_t_; Rh_t_; Q_t−1_	4-7-1	78.5	0.89	2.79	0.46	74.7	0.86	-5.14	0.50
04	R_t_; T_t_; Ws_t_; Q_t−1_	4-4-1	77.5	0.88	1.00	0.47	74.3	0.86	-3.38	0.57
**04**	**R**_**t**_; **R**_**t−1**_; **T**_**t**_; **Q**_**t−1**_	**4-8-1**	**81.5**	**0.91**	**1.01**	**0.43**	**79.3**	**0.84**	**-2.04**	**0.45**
05	R_t_; R_t−1_; T_t_; Ws_t_; Rh_t_	5-5-1	74.3	0.85	11.45	0.50	64	0.82	-10.63	0.59
05	R_t_; T_t_; Ws_t_; Rh_t_; Q_t−1_	5-7-1	83.3	0.91	1.31	0.40	74.1	0.88	-1.75	0.50
06	R_t_; T_t_; Ws_t_; Rh_t_; Q_t−1_; Q_t−2_	6-9-1	78.0	0.90	4.56	0.46	75.0	0.88	-2.12	0.49
06	R_t_; R_t−1_; T_t_; Ws_t_; Rh_t_; Q_t−1_	6-8-1	82.8	0.91	0.94	0.41	75.3	0.87	-2.90	0.49
07	R_t_; R_t−1_; R_t−2_; T_t_; Ws_t_; Rh_t_; Q_t−1_	7-9-1	83.3	0.93	3.26	0.41	74.2	0.88	-1.38	0.51
08	R_t_; R_t−1_; R_t−2_; T_t_; Ws_t_; Rh_t_; Q_t−1_; Q_t−2_	8-16-1	83.0	0.91	3.92	0.41	77.8	0.87	1.97	0.47

Where,

*R*_*t*_ rainfall, *R*_*t−1*_ rainfall one day before, *R*_*t−2*_ rainfall two days before, *T*_*t*_ temperature

*Ws*_*t*_ Wind speed, *Rh*_*t*_ relative humidity, *Q*_*t−1*_ runoff one day before, *Q*_*t−2*_ runoff two day before

**Table 8:** Performance evaluation of the ANN model during the training and testing period.

Neural Network	Training period				Testing period			
	NSE %	*R* ^2^	% Bias	RSR	NSE %	*R* ^2^	% Bias	RSR
4-4-1	79.9	0.80	2.42	0.44	75.5	0.80	-4.97	0.49
4-5-1	78.8	0.88	4.57	0.45	74.8	0.88	-3.59	0.50
4-6-1	80.8	0.89	3.03	0.43	75.3	0.89	-3.47	0.49
4-7-1	79.9	0.89	2.49	0.44	74.6	0.89	-4.69	0.50
**4-8-1**	**81.5**	**0.91**	**1.01**	**0.43**	**79.3**	**0.84**	**-2.04**	**0.45**

**Figure. 11:**
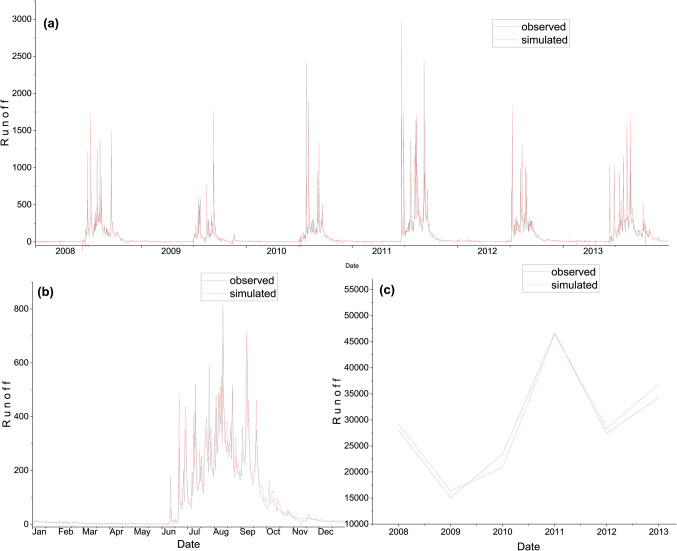
Comparison of observed and simulated runoff using ANN model at testing period. (**a**) daily; (**b**) average daily; (**c**) annual.

### ANN-based hybrid technique

**Figure. 12:**
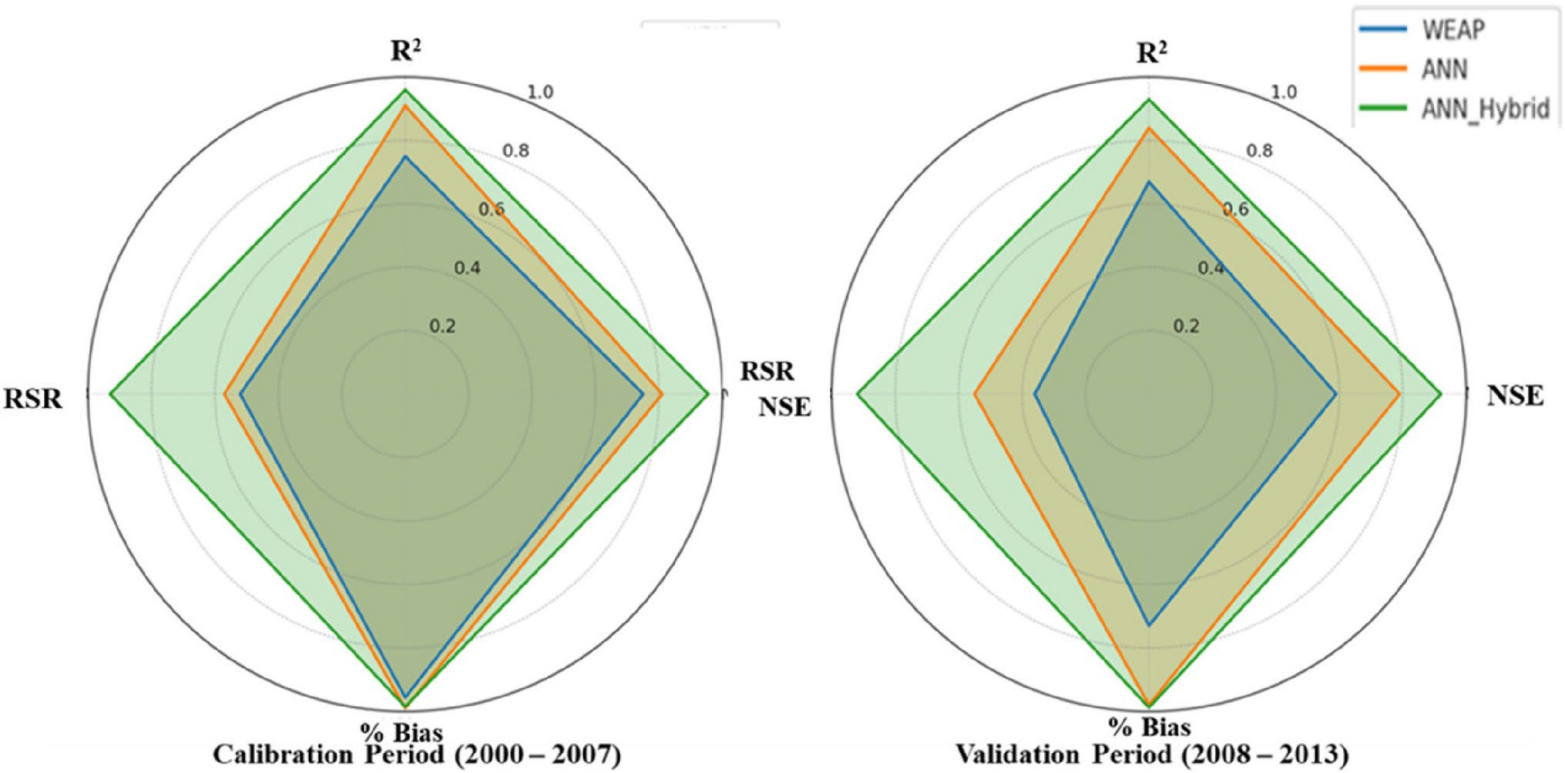
Web plot performance metrics of the WEAP, ANN and ANN_Hybrid_ model.

**Figure. 13:**
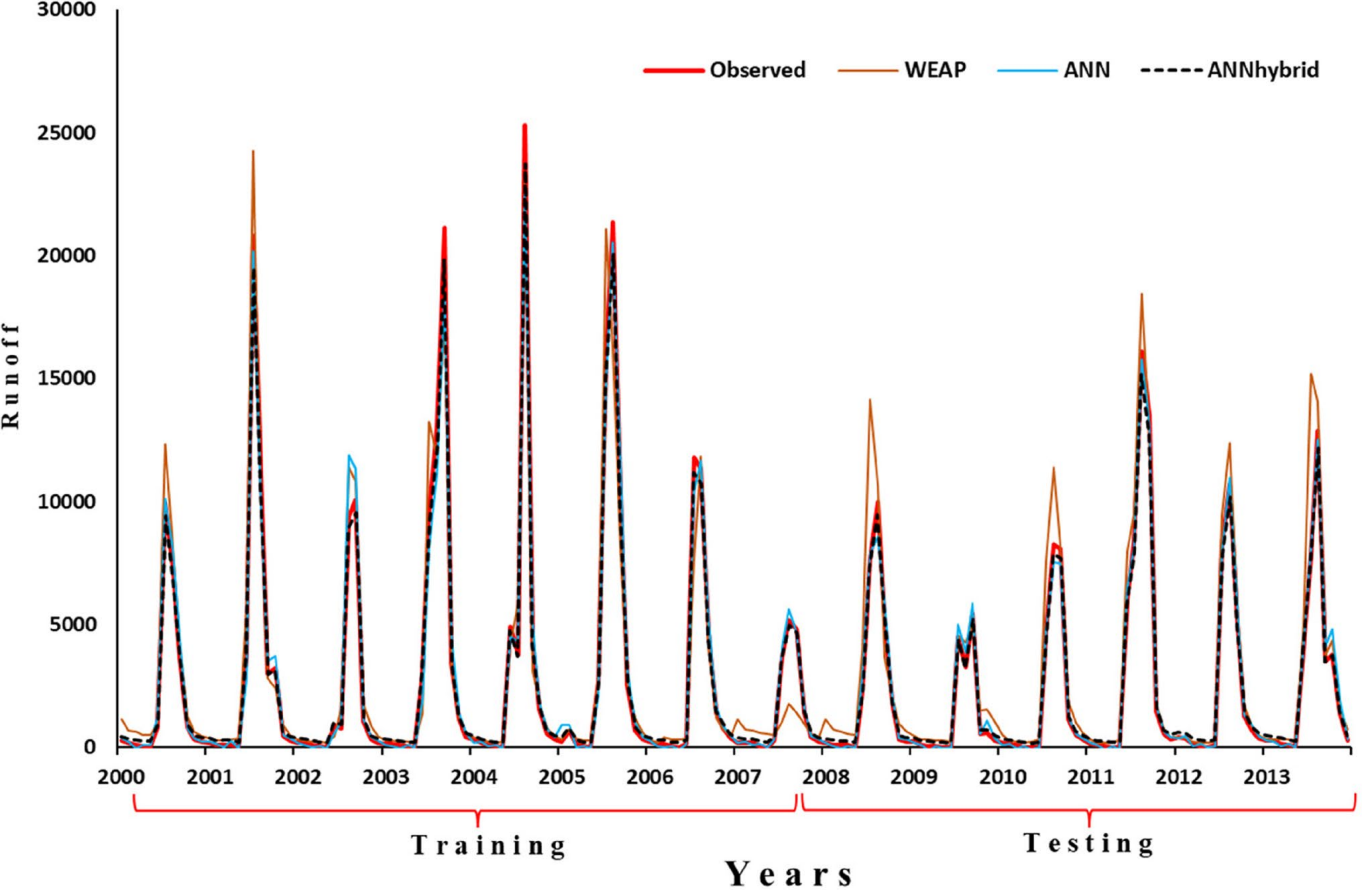
Comparison of observed and simulated runoff by WEAP; ANN & ANN_Hybrid_.

**Figure. 14:**
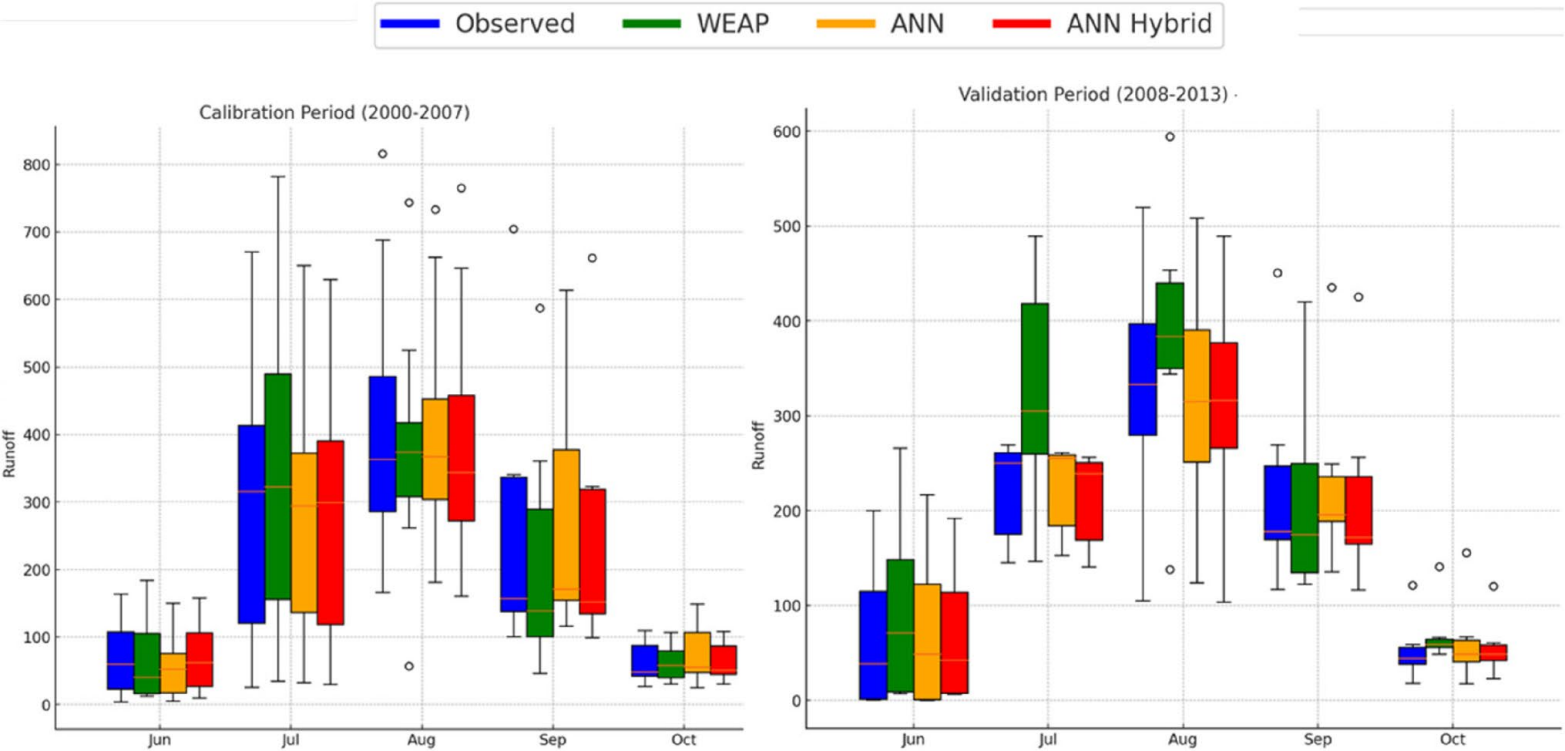
Box plot of comparison of observed with simulated monthly flow distribution in calibration and validation period.

The hybrid approach is a complex technique adopted to improve the accuracy of flow forecasts. The four-time series, explored by the ANN models from the previous section, and the one-time series, explored by the WEAP model for a total of five-time series, are adopted as inputs to a novel ANN in these experiments. The time series of the ANN models, namely the (R_t_, R_t−1_, T_t_, Q_t−1_), are the input to the optimal number of neurons results. Table 9 shows the comparison of performance evaluation indices for WEAP, ANN, and ANN_Hybrid_ model, whereas the web plot (Fig. 12) presents a visual comparison of the performance metrics for the WEAP, ANN, and ANN_Hybrid_ models during the calibration and validation periods. The ANN_Hybrid_ model exhibits the highest values for R^2^ and NSE and the lowest values for %Bias in both periods, indicating its superior performance compared to the standalone models. Further, the comparison of simulated daily runoff by WEAP, ANN and ANN_Hybrid_ models with the observed daily runoff during calibration/training and validation/testing period shown in Fig. 13. To further illustrate the performance of the models, box plots comparing the observed and simulated monthly flow distributions for monsoon months during the calibration and validation periods are presented in Fig. 14. The box plots demonstrate that the ANN_Hybrid_ model captures the monthly flow variability more accurately than the standalone WEAP and ANN models, particularly during the validation period, where the simulated flow distributions closely match the observed data.

**Table 9:** Comparison of performance of the WEAP and ANN model.

Performance criteria	Calibration/Training			Validation/Testing		
	WEAP	ANN	ANN_Hybrid_	WEAP	ANN	ANN_Hybrid_
R^2^	0.75	0.91	0.96	0.67	0.84	0.93
NSE	75%	81%	95.5%	59%	79%	0.92%
%Bias	-4.40	1.01	1.49	-27	-2.04	-1.22
RSR	0.48	0.43	0.07	0.64	0.45	0.08

## Discussions

The hydrological simulation of rainfall-runoff processes in the UNB using the WEAP and ANN models, and the novel ANN_Hybrid_ approach, not only advances our understanding of water resource management in this specific region but also demonstrates the transferability of these techniques to other hydrological applications and geographic locations. The input variables used in this study, such as rainfall, temperature, humidity, wind speed, and previous day’s runoff, are commonly measured variables that influence hydrological processes in many river basins worldwide^51,62^. By successfully integrating these variables into the modeling framework and improving the accuracy of streamflow predictions, our study showcases the versatility of the employed methods in capturing the complex interactions between atmospheric conditions, land surface characteristics, and runoff generation. Moreover, the ANN_Hybrid_ approach, which influences the strengths of both physically-based and data-driven models, offers a promising avenue for enhancing the performance of hydrological simulations in regions where data scarcity or process understanding may be limited^12,51^. The transferability of this hybrid approach to other basins and its potential to incorporate additional variables, such as solar radiation or land use change, highlights its value as a tool for sustainable water resource management and climate change adaptation strategies^62,63^. Thus, the novelty of our study lies not only in the specific application to the UNB but also in the demonstration of the broader applicability and impact of the employed modeling techniques for advancing hydrological research and water resource management practices in diverse settings.

The results achieved in this study have significant implications for streamflow prediction and water resource management in the UNB and similar data-scarce catchments. The proposed ANN_Hybrid_ model, which integrates simulated flow from both the physically-based WEAP model and the data-driven ANN model, demonstrates superior performance compared to the individual models. The hybrid approach leverages the strengths of both models, enabling more accurate and reliable streamflow simulations in the study area. Improved streamflow predictions are crucial for effective water resource planning, allocation, and management, especially in regions facing water scarcity and increasing water demands^53,64^. The UNB particular, plays a vital role in water provisioning for agricultural, domestic, and industrial uses in the region^11^. By providing more accurate streamflow estimates, the ANN_Hybrid_ model can support better decision-making related to water allocation, reservoir operations, and drought and flood risk assessment in the basin.

**Figure. 15:**
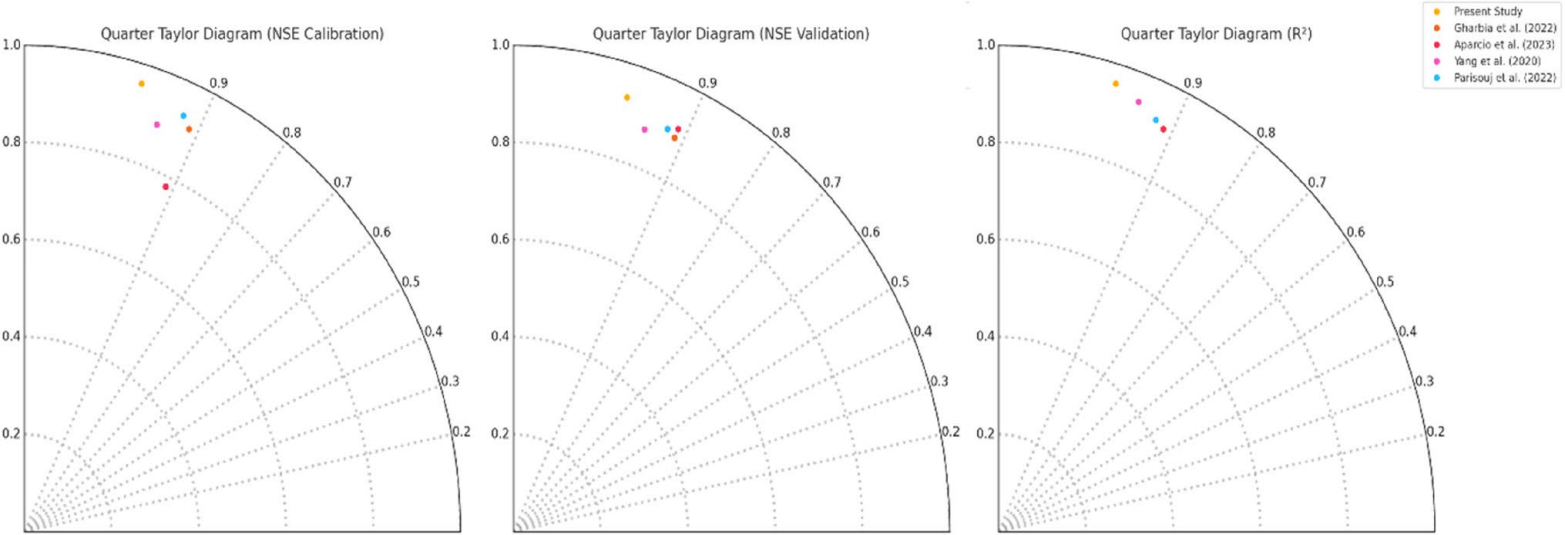
Comparative graph of present study with previous studies.

The present study demonstrates superior performance in streamflow simulation compared to similar studies conducted by^31,36,39,66^. As evident from the quarter Taylor diagrams shown in Fig. 15, our proposed ANN_Hybrid_ model exhibits the highest Nash-Sutcliffe efficiency (NSE) values of 0.96 and 0.93 during the calibration and validation periods, respectively, along with an impressive R^2^ value of 0.96. These metrics indicate that our model captures the observed streamflow patterns with exceptional accuracy and minimal residual variance. In comparison, the HEC-HMS-LSTM model developed by^31^ achieved lower NSE values of 0.93 and 0.90, with an R^2^ of 0.92, suggesting that their model, while performing well, may not capture the streamflow dynamics as precisely as our ANN_Hybrid_ model. Similarly^36^, GBHM-ANN-CA-CV model obtained NSE values of 0.89 and 0.88 and an R^2^ of 0.94 during the same periods, indicating that their approach, although effective, may not provide the same level of accuracy as our proposed model. The wavelet-ANN (WANN) model by^39^ showed NSE values of 0.91 and 0.89, with an R^2^ of 0.91, which, while commendable, still falls short of the performance achieved by our ANN_Hybrid_ model. Lastly, the best-performing model by^66^ reached NSE values of 0.78 and 0.91 for calibration and validation, respectively, with an R^2^ of 0.92, suggesting that their approach may not consistently capture the streamflow dynamics across both periods as effectively as our model.

The superior performance of our ANN_Hybrid_ model can be attributed to several key factors. Firstly, the effective integration of physically-based and data-driven approaches leverages the strengths of both methodologies, enabling our model to capture the complex rainfall-runoff relationships more accurately than the standalone models used in the comparative studies. By combining the hydrological knowledge embedded in the WEAP model with the learning capabilities of the ANN, our proposed approach can better represent the underlying physical processes while also adapting to the unique characteristics of the study area. The methodological advancements set our study apart from the previous works and establish its credibility in the field of streamflow modeling. The superior performance of our ANN_Hybrid_ model, as demonstrated by the higher NSE and R^2^ values, highlights its ability to reliably simulate streamflow in the study area. The robustness and versatility of our approach suggest that it could be applied to other watersheds with different hydrological characteristics, making it a valuable tool for water resource management and planning.

The proposed hybrid ANN model offers a practical and reliable tool for streamflow prediction in the UNB. Its superior performance and computational efficiency can support informed decision-making for water resource management, flood risk assessment, and hydraulic engineering design^39,65,67,67^. The findings of this research have the potential to benefit engineers, water managers, and stakeholders in the region by providing them with an accurate and efficient means of simulating streamflow, ultimately contributing to sustainable water resource management and resilience against future challenges.

The ANN_Hybrid_ model’s performance relies on the quality and quantity of input data, which were obtained from limited monitoring stations that may not fully capture the spatial variability of the hydro-meteorological processes in the UNB. However, the successful application of the hybrid approach demonstrates its potential to enhance streamflow simulations in regions where long-term hydrological observations are scarce, providing a foundation for future research on hybrid modeling approaches and their application in water resource management.

Future scope: Furthermore, the methodology presented in this study can be extended to other data-limited catchments facing similar challenges in streamflow prediction. The successful application of the hybrid approach in the UNB demonstrates its potential to enhance streamflow simulations in regions where long-term hydrological observations are scarce^49^. The improved predictions can ultimately contribute to more sustainable and efficient water resource management practices in these areas.

## Conclusions

In this study, we proposed a novel hybrid approach, ANN_Hybrid_, that combines a physically-based hydrological model (WEAP) with a data-driven model (ANN) to enhance streamflow prediction accuracy in the data-scarce UNB. The results demonstrate that the ANN_Hybrid_ model outperforms the standalone WEAP and ANN models in simulating streamflow, particularly during the training and testing periods. The superior performance of the ANN_Hybrid_ model can be attributed to its ability to leverage the strengths of both the physically-based and data-driven approaches. By integrating the hydrological knowledge embedded in the WEAP model with the learning capabilities of the ANN, the hybrid approach captures the complex rainfall-runoff relationships more accurately than the individual models. This is evidenced by the higher Nash-Sutcliffe efficiency (NSE) values achieved by the ANN_Hybrid_ model compared to the WEAP and ANN models during both the calibration (95.5% vs. 75% and 81%, respectively) and validation (92.3% vs. 59% and 79%, respectively) periods.

The improved streamflow predictions provided by the ANN_Hybrid_ model have significant implications for water resource management and planning in the UNB. The enhanced accuracy and reliability of the simulations can support better decision-making related to water allocation, reservoir operations, and flood and drought risk assessment. These improvements are particularly crucial in regions facing water scarcity and increasing water demands, such as the study area. Furthermore, the methodology presented in this study can be extended to other data-limited catchments facing similar challenges in streamflow prediction. The successful application of the hybrid approach in the UNB demonstrates its potential to enhance streamflow simulations in regions where long-term hydrological observations are scarce. By combining the strengths of physically-based models with the flexibility and learning capabilities of data-driven techniques, the proposed ANN_Hybrid_ approach offers a promising avenue for improving hydrological predictions in data-scarce environments.

In conclusion, this study contributes to the advancement of hydrological modeling by demonstrating the effectiveness of combining physically-based and data-driven models for improved streamflow predictions in data-scarce regions. The proposed ANN_Hybrid_ model offers a practical and reliable tool for supporting sustainable water management and climate change adaptation strategies in the UNB and other similar catchments. The findings provide a foundation for future research on hybrid modeling approaches and their application in water resource management, particularly in regions where data limitations and complex hydrological processes pose challenges for accurate streamflow prediction.

## Data Availability

The datasets used and/or analysed during the current study available from the corresponding author on reasonable request.
